# Evaluating the efficiency of emergency operation centers during pandemics: a cross-sectional study with operational managers in Iran

**DOI:** 10.3389/fpubh.2025.1511932

**Published:** 2025-05-30

**Authors:** Majid Sartipi, Asiye Aminafshar, Abdolrazzagh Pakzad, Mohammadreza Shafiei, Hojjat Farahmandnia, Asghar Tavan

**Affiliations:** ^1^Infectious Diseases and Tropical Medicine Research Center, Research Institute of Cellular and Molecular Sciences in Infectious Diseases, Zahedan University of Medical Sciences, Zahedan, Iran; ^2^Department of Biostatistics and Epidemiology, School of Health, Zahedan University of Medical Sciences, Zahedan, Iran; ^3^Student Research Committee, Kerman University of Medical Sciences, Kerman, Iran; ^4^Health Promotion Research Center, Zahedan University of Medical Sciences, Zahedan, Iran; ^5^Health, Safety, and Environmental Management (HSE), Zahedan University of Medical Sciences, Zahedan, Iran; ^6^Department of Nursing, School of Nursing, Larestan University of Medical Sciences, Larestan, Iran; ^7^Health in Disasters and Emergencies Research Center, Institute for Futures Studies in Health, Kerman University of Medical Sciences, Kerman, Iran

**Keywords:** efficiency, incident command system, pandemic, EOC, PCA

## Abstract

**Background:**

An effective crisis management system like the Emergency Operations Center (EOC) influences the number of damages and human casualties during disasters and pandemics. This study examined the preparedness and management functions of the Zahedan University of Medical Sciences incident command system (ICS) in response to incidents and disasters, focusing on its implementation during the pandemic.

**Materials and methods:**

The study employed a cross-sectional design. All members of the university’s ICS at various levels were included in the study. The data collection tool was a researcher-made questionnaire. The PCA (Principal component analysis) method was employed in SPSS 27 statistical software for data analysis.

**Results:**

Among the extracted components, the highest mean was associated with the *command and empowerment of operational teams* (C2), with a mean of 3.72 (CI: 3.04–4.40). The managers working in the crisis management headquarters, at the planning and comprehensive command level, performed better than other management levels. A significant relationship was found between past crisis experience and the scores of all extracted components. Individuals who had previously been involved in crises scored higher. The *p*-values for components 1 to 4 were 0.002, 0.001, 0.005, and 0.019, respectively. In C1 (Comprehensive risk planning) and C2, individuals with higher education obtained better scores, 3.85 (CI: 3.15–4.55) and 3.92 (CI: 3.22–4.62) compared to individuals with lower education 3.46 (CI: 2.79–4.13) and 3.57 (CI: 2.93–4.21) (*p*-values 0.011 and 0.02, respectively). Also, continuous training significantly helped improve the performance of managers. Other variables such as field of study, type of employment, and job history did not show significant differences.

**Conclusion:**

Past experiences significantly impact people’s ability to prepare and make quick decisions in times of crisis. It is essential to provide effective solutions to future managers and implement corrective measures within the crisis management system. Additionally, training and empowerment programs should be planned for all healthcare workers, and greater attention should be paid to investing in young forces.

## Introduction

Natural and man-made disasters have had considerable health effects on the communities. In 2021, 432 natural disasters were recorded worldwide, a number that is increasing globally. In recent decades, pandemics have resulted in widespread disasters (1). Infectious disease pandemics are among the most significant health threats. A pandemic is a widespread infectious disease that crosses international borders and affects a large number of people in multiple regions or continents (2). In addition to mortality, viral and bacterial pandemics have adverse economic, social, and health effects on societies (3). Weaknesses in infrastructure, a lack of resources, and inadequate coordination between different institutions are among the primary factors that contribute to the complexity and prolongation of crises, including the management of pandemics (4). We require effective management to mitigate the impacts of pandemics. We must rebuild the emergency health system, establish emergency health departments, strengthen international and domestic cooperation, and strengthen public interventions in response to public health crises and pandemics (5).

In Iran, the number of disasters increased from fewer than 100 cases per year before 1980 to 300 to 400 cases between 2000 and 2019. Considering the frequency of occurrence, extent, and population density, these risks lead to both small and large disasters that sometimes require difficult recovery (6, 7). An incident with moderate severity can also affect the country’s healthcare system (8). One goal of responding to incidents and disasters in the healthcare system is to prevent and reduce casualties and physical and mental injuries. This can only be achieved with careful planning, preparation, and training of the human resources involved in the crisis (9, 10).

On the other hand, managers need access to coherent, integrated, accessible, and coordinated health services. A lack of proper planning, resource management, and intersectoral coordination will challenge the provision of healthcare services during disasters (11–13). Integrated disaster management at the global level requires a conceptual definition, such as Emergency Operations Centers (EOCs). These centers have various departments, including planning, operations, logistics, and information and communication technology (14). Strategic decisions for disaster management, coordination of multilateral responses to health incidents, collection and distribution of resources in the emergency operations center, timely information announcement to the public and the media, and interagency coordination are made here. The correct functioning of these centers enables the efficient allocation and distribution of resources, including human resources, equipment, and supplies, which must be directed to the areas with the greatest need (15).

Additionally, EOCs serve as command-and-control centers to coordinate emergency responses, facilitate risk assessment, and manage critical information. With the integration of an Incident Management System (IMS), these centers serve as strategic tools for disaster management. Standardizing processes and improving coordination help enhance incident preparedness and response (16). In addition to incidents and disasters, EOCs possess the necessary capacity to manage infectious diseases during pandemics. The World Health Organization (WHO) has also recognized the importance of EOCs in dealing with epidemics and has provided guidelines for establishing these centers at the national level. WHO recommends that Member States develop and strengthen the necessary infrastructure for managing health crises, including the establishment and strengthening of EOCs, so that they can respond effectively and in a coordinated manner to large outbreaks (17). The widespread outbreak of Ebola in West Africa between 2014 and 2016 drew the attention of policymakers to the critical role of emergency operations centers in managing health crises in countries with limited resources (18). Studies by Shuaib et al. (2017) and OO Olu et al. (2016) demonstrated that these centers can serve as effective tools in controlling the spread of diseases in these areas (16, 19). Emergency Operations Centers (EOCs) play a crucial role in managing health emergencies, particularly in developing countries with limited infrastructure. Case studies in regions such as Nigeria, Southeast Asia, and Africa have demonstrated the effectiveness of these centers in controlling infectious diseases, including Ebola, polio, COVID-19, and dengue fever (16, 20, 21). These centers enhance emergency response, mitigate the negative impacts of disasters, and expedite decision-making by coordinating efforts among various organizations and stakeholders. They provide monitoring tools and data analysis, collect, analyze, and disseminate information. Additionally, EOCs effectively manage resource constraints and improve health outcomes in resource-limited countries by facilitating resource allocation, strengthening collaboration and data sharing, and enhancing response times (16, 18, 21–23).

Early activation of the Public Health Emergency Operations Center (PH-EOC) and the use of an incident management system (IMS) during the dengue outbreak in Pakistan resulted in providing an organizational structure with clear roles and tasks for different teams, a transparent chain of Command, and an interdepartmental coordination mechanism. And the integration of accountability activities. This resulted in the early control of dengue outbreaks compared to previous outbreaks. Also, human resources training and EOC standby activation are necessary during non-pandemic periods to implement IMS in the shortest possible time. With the adoption of IMS, more effective planning, more robust coordinated response, and improved communication with health authorities and the general public will be achieved (23). The H1N1 influenza pandemic in 2009 highlighted the importance of interdepartmental coordination and information management in EOCs (24) Obstacles and factors, including IMS, communication strategies, data management, workforce capacity, and physical infrastructure, affect the effective use of these centers in managing public health emergencies (25). In their study, Tsukayama et al. (2023) examined the coordination between Public Health Emergency Operations Centers (PHEOC) in Thailand, Cambodia, Laos, and Malaysia. They found that each of these countries is committed to strengthening its national centers and enhancing cross-border communication in response to the COVID-19 pandemic. Still, gaps remain in data sharing, workforce capacity, and use of communication platforms (25).

Ryan et al. (26) investigated the importance of planning in EOCs. They stated that detailed and comprehensive planning in these centers is vital for the success of field operations and for maintaining situational awareness, especially in disaster situations. This article states that limited timing and incomplete information can create significant challenges in planning, but having a comprehensive plan can improve the accuracy and efficiency of the response. EOCs play a vital role in disaster response, and therefore, the continuity of services in disaster management is crucial for EOCs. Still, these centers may face challenges that disrupt continuity in decision-making (25, 27).

K.S-Małyjurek et al. (28) investigated the challenges in coordination between different organizations in EOCs. They demonstrated that issues such as high information load and inter-organizational inconsistency can impact the continuity of services during emergencies and disasters. This study proposes strategies to enhance coordination and improve information management, including strengthening coordination and information management within EOCs to ensure the continuity of services.

During the last global pandemic, COVID-19, EOCs played a crucial role in facilitating pandemic management. Following the World Health Organization’s announcement of the COVID-19 pandemic, Iran was also affected on February 19, 2020. By the end of October 2024, the number of confirmed COVID-19 patients in Iran was about 7,627,186, and the number of deaths was about 164,000 (29). According to the database of Zahedan University of Medical Sciences (ZUMS), since the beginning of the COVID-19 pandemic worldwide, Sistan and Baluchistan province has experienced three waves of disease outbreaks: in June 2020, October 2020, and May 2021. These statistics show that COVID-19 has had a broad impact on Iranian society, and a significant number of people have been affected by this disease (30).

Indeed, effective management in EOCs plays a crucial role in controlling crises and pandemics, as well as coordinating and enhancing the efficiency of measures. Since managers’ success in incident management depends on proper performance during disasters, it is necessary to identify the vulnerable points of the health system in disaster management to intervene effectively and improve performance as a result. Considering the importance of the incident command center’s efficiency in responding to disasters and incidents, this study aims to evaluate the effectiveness of the IMS at ZUMS and Health Services, one of the key response organizations in incidents and disasters during pandemic conditions.

## Materials and methods

The current research was a cross-sectional study that investigated the efficiency of Zahedan University of Medical Sciences’ incident management system in 2019 in responding to the pandemic. The study population of all the members of the research community, i.e., managers of the university incident command and operational managers, based on the different departments of the EOC of the university and its subordinate cities, was expected to be 110 people, which included all the managers working in the university. The sampling method employed was a census, and all EOC managers, including senior, middle, and operational levels, were invited to participate in the study. However, 82 people cooperated to complete the evaluation questionnaire despite trying to maximize the number of participants. The questionnaire for managers working in Zahedan City was completed in person, while for other cities, the questionnaire was emailed, and participants were guided by phone coordination. After completing the questionnaires, we received them by post. The criteria for inclusion in the study was membership in the incident command system, and the exclusion requirement was the unwillingness to participate.

The data collection tool was a questionnaire prepared based on the national checklist for evaluating managerial functions in the preparation and response phases, as outlined in the national program for the health system’s response to disasters and emergencies, as notified by the Ministry of Health, Treatment, and Medical Education of Iran. Two indexes, the content validity index (CVI) and the content validity ratio (CVR), were employed to assess the content validity of the research tool. The validity of the questionnaire was evaluated by sending it to 10 health experts in disaster management, and their correction comments were incorporated into the final questionnaire. The CVI value obtained was 0.85, indicating strong content validity. Additionally, a CVR value of 0.70 was calculated, indicating the need to ask questions based on experts’ opinions. The reliability of the questionnaire was measured through a panel of experts and using Cronbach’s alpha coefficient (*α* = 0.89).

The questionnaire included a demographic information section with nine questions to collect data, and 80 questions were evaluated to measure the functions of the Emergency Operation Plan (EOP). The main questions were scored using a Likert scale of “very poor,” “poor,” “average,” “good,” and “excellent.” The total score of the main questions was categorized between 80 and 400. All the questionnaire questions are listed in Table 1.

**Table 1:** Components and coefficients of each of the study indices (questions), extracted from PCA in participants of the incident management system of Zahedan University of Medical Sciences.

No.	Items	Components			
		C1	C2	C3	C4
1	Development of monitoring and evaluation program	0.801	0.206	0.127	0.306
2	Having a plan to collect and summarize general health and safety advice for at-risk populations	0.796	0.218	0.216	0.229
3	The existence of a process for receiving, analyzing and sending news to the media	0.794	0.283	0.168	0.204
4	Determining the interviewer with the media as well as the content of the interviews	0.742	0.266	0.247	0.161
5	Collect reports of all units	0.732	0.284	0.123	0.274
6	Monitoring related news in the media	0.731	0.213	0.076	0.415
7	The existence of an evacuation officer in each department/ unit	0.723	0.208	0.405	0.015
8	The existence of a list of approved mass media for information exchange	0.721	0.33	0.304	0.098
9	Supervising the formulation of the evacuation plan for subordinate units	0.721	0.277	0.35	0.188
10	Providing feedback on evaluation results to units	0.713	0.385	0.046	0.201
11	Having an evacuation plan for hospitals and health units	0.695	0.275	0.445	0.095
12	Determining the weaknesses and strengths of program implementation	0.683	0.324	0.158	0.299
13	Summarizing the results of the investigation and presenting a report to the superior officer	0.66	0.493	−0.004	0.203
14	Cooperation and interaction with the police/military/inactive defence organization	0.655	0.323	0.35	0.132
15	The readiness of the personnel gathering place	0.624	0.058	0.454	0.152
16	Monitoring the opening of evacuation routes	0.594	0.137	0.496	0.072
17	Determining the locations of physical protection forces	0.587	0.382	0.429	0.091
18	Communicating the compiled Incident Action Plan (IAP) to all operational units	0.578	0.232	0.431	0.066
19	The existence of an evacuation notification system	0.567	0.227	0.473	0.05
20	The existence of a specific command successor	0.56	0.476	0.338	−0.064
21	Controlling the traffic of people and cars at the entry and exit points	0.554	0.39	0.355	0.105
22	Upgrade existing programs based on evaluation results	0.543	0.45	0.052	0.194
23	Compliance with the principle of single Command (everyone has only one superior)	0.524	0.438	0.408	0.04
24	Monitoring and ensuring the continuity of service delivery	0.505	0.087	0.443	0.332
25	Developing a specific protocol for the security of sensitive and essential units (the presence of a program to control the entry of clients, protect personnel, control entrances and exits, etc.)	0.489	0.354	0.463	0.034
26	The existence of a specific process for sending orders	0.468	0.384	0.408	0.07
27	Statistics of security needs	0.461	0.216	0.444	0.086
28	Ensuring activation of the joint rapid assessment process	0.46	0.454	0.364	0.267
29	Communicate with the Joint Rapid Assessment Team based on the status quo	0.443	0.435	0.27	0.298
30	The familiarity of the command team members with IAP	0.443	0.434	0.36	0.122
31	Requesting the evaluation team’s report and preliminary analysis of the report	0.429	0.392	0.205	0.291
32	Evaluating the effectiveness of training	0.19	0.731	0.24	0.105
33	The existence of educational ID cards for personnel	0.36	0.722	0.245	−0.02
34	Planning is based on EOP	0.356	0.719	0.158	0.188
35	Implementation of the training program according to the set schedule	0.152	0.704	0.415	0.008
36	The existence of EOP compiled	0.337	0.692	0.129	0.251
37	Exercise based on EOP	0.319	0.674	0.113	0.182
38	Ensuring the presence of command elements at the scene command post	0.175	0.666	0.206	0.245
39	Ensuring timely activation of the command post in the affected area	0.269	0.651	0.149	0.271
40	Compilation and presentation of risk assessment report	0.206	0.626	0.114	0.404
41	Announcing the news and warning level to the cooperating units and the operational team	0.36	0.624	0.182	0.353
42	Communication with the Emergency Operations Center (EOC)	0.498	0.614	0.22	−0.058
43	Collect and analyze risk assessment data	0.311	0.612	0.039	0.475
44	Ensuring the activation of the force recall process	0.085	0.597	0.348	0.212
45	Development of scenarios and exercise evaluation protocol	0.34	0.584	0.377	0.012
46	Receive news 24 h a day from risk monitoring centres (before and during an incident)	0.416	0.582	0.008	0.316
47	Compilation of disaster management training matrix in such a way that it is determined which category of employees should be trained for which subject	0.244	0.578	0.346	0.125
48	The familiarity of the command team members with the role of the command staff in the exercise	0.465	0.576	0.316	−0.021
49	Notification and alert level to upper levels (MOH-EOC)	0.355	0.561	0.213	0.353
50	Communicating with the coordination chief for the call of force from other organizations	0.099	0.555	0.295	0.288
51	Dissemination of information to target groups (senior public relations, operations, etc.)	0.535	0.542	0.26	0.113
52	Formulating the training program in such a way that it is determined which category of managers and employees should be trained for which subject	0.292	0.539	0.454	0.177
53	Existence of task descriptions for the command team (tasks of people in the crisis room are divided, and people know how to perform these tasks)	0.404	0.526	0.38	−0.073
54	Familiarity of command team members with ICS	0.477	0.524	0.307	0.037
55	Timely activation of ICS	0.365	0.51	0.408	−0.021
56	Monitoring the use of personal protective equipment	0.384	0.483	0.414	−0.155
57	Continuous monitoring of environmental data and their analysis (SitRep)	0.247	0.482	0.36	0.005
58	Participation in manoeuvre programs and exercises of other organizations	0.298	0.479	0.417	0.226
59	Predicting the list of needed supplies and necessities	0.047	0.449	0.417	0.447
60	The presence of supplies and equipment needed to communicate between units	0.192	0.178	0.829	0.172
61	The existence of multi-layered communication platforms between operational units	0.233	0.194	0.734	0.191
62	The existence of a communication program between units	0.466	0.148	0.715	0.167
63	Compilation of protocols related to forecasting equipment and supplies	0.062	0.462	0.651	0.227
64	Development and communication of safety protocols	0.263	0.182	0.632	0.165
65	Provision of patient transportation and equipment	0.305	0.175	0.629	0.266
66	Ensuring the assessment of the safety situation of the area and documenting it	0.328	0.285	0.614	0.112
67	Periodic control of stocks	−0.063	0.431	0.614	0.244
68	Supplying supplies and equipment to provide services as requested	0.307	0.259	0.603	0.26
69	Providing a safe and comfortable service environment in the headquarters and stage (ICP)	0.414	0.27	0.585	0.205
70	Implementation of the exercise program based on needs assessment and review of lessons learned from previous exercises	0.193	0.515	0.54	0.144
71	Provision of critical infrastructure and routes in the headquarters and the scene	0.334	0.196	0.515	0.336
72	Formulation of IAP based on EOP	0.446	0.324	0.509	0.202
73	Distribution of items based on safety protocols	0.401	0.326	0.443	−0.11
74	Evaluating memorandums of understanding	0.21	0.125	0.086	0.841
75	Continuous monitoring of the provisions of the memorandum and its review	0.208	0.058	0.161	0.785
76	Applying the proposal, amending the terms of the memorandum	0.086	0.118	0.177	0.763
77	Drafting a memorandum of cooperation in which the roles and responsibilities are clearly stated	0.128	0.105	0.191	0.757
78	Conducting a risk assessment of healthcare facilities annually	0.377	0.362	−0.006	0.644
79	Analysis and identification of beneficiaries for each general and specific function	0.194	0.188	0.337	0.606
80	Pursuing the equipping of health centers, health houses and operational units	−0.024	0.213	0.454	0.505

Extraction method: Principal component analysis.

Rotation method: varimax with Kaiser Normalization.

**Figure 1:**
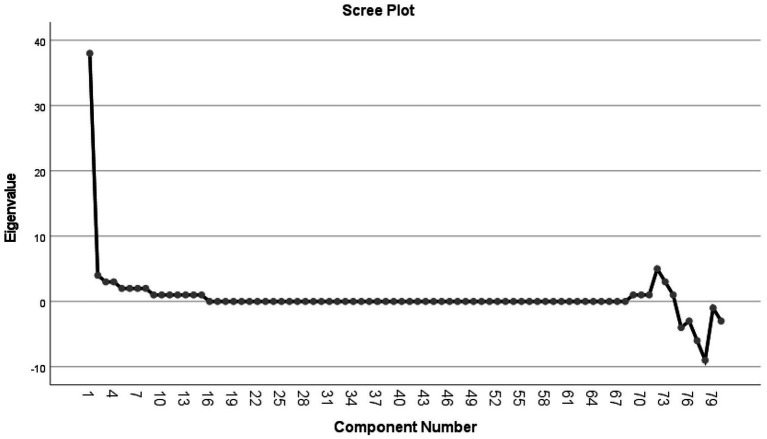
Scree plot of the component number and eigenvalues of questions based on PCA in participants of the incident management system of Zahedan University of Medical Sciences.

Due to the large number of questions in the questionnaire and the inability to perform applied analyses, such as regression models, directly on these questions, it was necessary to extract latent variables from the data. For this reason, analysis methods that reduce the dimensions of the data, such as factor analysis, had to be used; therefore, the Principal Component Analysis method was used to reduce the dimensions of the data. PCA is used to reduce the dimensions of many variables (questions) related to each other. PCA primarily aims to replace many correlated variables with uncorrelated principal components in regression models, thereby mitigating concerns about collinearity. The model’s first few principal components account for the most significant proportion of the total variance. The rationale for using PCA is to reduce the data’s complexity, increase the accuracy of the analysis by reducing information noise, and identify structures and patterns that may not be easily observed (31). To perform this analysis, first, KMO and Bartlett’s tests were performed for the possibility of PCA, which, according to the value of KMO = 0.907, was higher than 0.5 (32). The value of Bartlett’s statistic is 8253.894 with 2,628 degrees of freedom and a *p* value of less than 0.001, indicating a significant correlation between the questions. This correlation is large enough for PCA, and our data are deemed suitable for performing PCA. Then, based on the screen plot, where the point of curvature was detected in component number 4, and the total variance table was explained, the number of 4 components with a total variance of 62.888% of the total Variance of the variables (questions) explained by them, remained in the analysis (Figure 1; Table 2). Sometimes, there are defects in orthogonal models, such as non-uniformity of the explained variance distribution (so that the most considerable variance is related to the first component); there is a relationship between the variables with more than one component, so that cannot be identified the main component related variable and the coefficients of the variables are not close to 1 (high impact of the component on the variable) or zero (low impact of the component on the variable). In this case, different rotation methods will be used, the most common of which is varimax, which will solve the mentioned problems to a large extent (31). Therefore, using Varimax rotation, the coefficients of each question were determined in four components (Table 1). Then, by consulting the relevant experts, these components were named based on the questions that had the highest coefficients in each component. The questions of these components were named as follows: 31 questions in the first component (C1) with the name of *Comprehensive risk planning*, 28 questions in the second component (C2) under the title of *Command and empowerment of operational teams*, 14 questions in the third component (C3) with the name of *Support and continuity of service* and seven questions for the fourth component (C4) under the title of *Developing and strengthening inter-sectoral cooperation*. This study analyzed the components as scores and compared them to other variables, such as gender and education. Therefore, the ANOVA method was used to compare the average scores of the extracted components across different categories of measured variables. One of the assumptions of ANOVA is that the variance components in various categories of comparable variables are homogeneous. If this homogeneity of variances was not established in different classes, the Kruskal-Wallis method was used. SPSS 27 software was used for data analysis, with a significance level 0.05 in all tests.

**Table 2:** The total variance explained extracted from PCA in participants of the incident management system of Zahedan University of Medical Sciences (Up to component 20 for brevity).

Total variance explained									
Components	Initial eigenvalues			Extraction sums of squared loadings			Rotation sums of squared loadings		
	Total	% of variance	Cumulative %	Total	% of variance	Cumulative %	Total	% of variance	Cumulative %
1	38.801	48.502	48.502	38.801	48.502	48.502	16.763	20.954	20.954
2	4.165	5.207	53.708	4.165	5.207	53.708	14.945	18.681	39.635
3	3.788	4.736	58.444	3.788	4.736	58.444	11.803	14.753	54.388
4	3.555	4.444	62.888	3.555	4.444	62.888	6.800	8.500	62.888
5	2.826	3.533	66.420						
6	2.542	3.178	69.598						
7	2.177	2.721	72.319						
8	2.032	2.540	74.859						
9	1.705	2.131	76.990						
10	1.463	1.828	78.818						
11	1.343	1.678	80.496						
12	1.282	1.603	82.099						
13	1.215	1.519	83.618						
14	1.079	1.348	84.967						
15	1.030	1.287	86.254						
16	0.870	1.087	87.341						
17	0.757	0.947	88.288						
18	0.735	0.919	89.207						
19	0.691	0.864	90.071						
20	0.636	0.795	90.866						

Extraction method: principal component analysis.

## Results

### Demographic

This study included 82 senior, middle, and operational managers from the university’s Incident Command System (ICS). The average age of these managers was 40.9 years, with a standard deviation of 7.96 years. Twenty women (24.4%) and 62 men (75.6%) participated. The education of the participants was as follows: 46 (56.1%) were bachelor’s degree holders, 20 (24.4%) were master’s degree holders, 8 (9.8%) were general practitioners, 2 (2.4%) PhD, 5 (6.1%) were clinical specialists, and 1 (1.2%) was a subspecialist. Additionally, in terms of field of study, 36 people (43.9%) studied health, 27 people (32.9%) studied medicine and paramedicine, and 19 people (23.2%) studied the humanities. Of the participants in the study, 47 individuals (57.3%) worked in the district health network, 30 individuals (36.6%) worked in hospitals, and five individuals (6.1%) worked in the crisis command headquarters. The working experience of managers ranged from 3 to 38 years, with a standard deviation of 8.8 years. The employment status of 43 people (52.4%) was official, 21 people (25.6%) held limited-term contracts, and 18 people (22.0%) were employed on a contract basis. Twenty-three managers (28.0%) did not have the experience of being in any crisis; 20 of them (24.4%) were present in only one crisis, and 39 (47.6%) experienced more than one crisis. Among the managers participating in the study, 80 people (97.6%) had participated in crisis management training courses. Of these, 40 people (48.8%) had participated in one course, and the remaining 40 had participated in more than one course. The managerial titles of the people participating in the study are listed in Table 3. The highest frequency was associated with health operations managers (18.3%), who were crucial in responding to crises and managing healthcare services.

**Table 3:** The distribution of the organizational posts of managers in participants of the incident management system of Zahedan University of Medical Sciences.

Management title	Frequency	Percent	Cumulative percent
Commander	10	12.2	12.2
Senior Coordination	8	9.8	22.0
Senior Communication	3	3.7	25.6
Senior Security Officer	9	11.0	36.6
Senior Safety Officer	7	8.5	45.1
Financial Support	10	12.2	57.3
Medicines and equipment	3	3.7	61.0
planning	8	9.8	70.7
Treatment operation	9	11.0	81.7
Health operations	15	18.3	100.0
Total	82	100.0	

### PCA results

The average, standard deviation, minimum, and maximum scores obtained in the four components by the studied managers are listed in Table 4. The highest average score was related to the *Command and empowerment of operational teams* (C2), with an average of 3.72 (CI: 3.04–4.40). The component of *Developing and strengthening inter-sectoral cooperation* (C4) had the lowest average among the components, with a mean of 3.41 (CI: 2.61–4.21). The standard deviation of 0.80 also indicated significant differences in managers’ ability to develop and strengthen interdepartmental cooperation.

**Table 4:** The distribution of average, standard deviation, minimum, and maximum scores by four components based on PCA in participants of the incident management system of Zahedan University of Medical Sciences.

Components		C1	C2	C3	C4
Number	Valid	82	82	82	82
	Missing	0	0	0	0
Mean		3.63	3.72	3.62	3.41
Std. Deviation		0.71	0.68	0.68	0.80
Minimum		1.48	2.25	1.71	1.14
Maximum		5.00	5.00	5.00	5.00

### ANOVA/Kruskal-Wallis findings

In the analysis of Variance (ANOVA), the average answers of managers at the three levels of the crisis management headquarters, the health network, and hospitals to the questions in the first three components had significant differences. Still, this difference was not significant in C4. The managers of the crisis management headquarters demonstrated a better performance in *Comprehensive risk planning* (C1). Managers at different management levels performed differently in C2. The managers of the crisis management headquarters performed better in the Command. The average scores for the third component differed across management levels. No significant difference was observed in C4, which shows that all management levels generally performed similarly in developing interdepartmental cooperation (Table 5). Additionally, the average responses of managers, categorized by those holding a bachelor’s degree or higher, in components 1 and 2, showed significant differences.

**Table 5:** Mean distribution and analysis of variance of the difference between the mean scores of questions related to management hierarchy by four components based on PCA in participants of the incident management system of Zahedan University of Medical Sciences.

Descriptives						ANOVA						
Components	*N*	Mean	Std. deviation	Std. error	Components	Sum of squares	df	Mean square	*F*	Sig.	Components	*N*
C1	UDMH*	5	4.5	0.76	0.3	C1	Between Groups	6.31	2	3.15	7.2	0.001
	DHN**	47	3.4	0.69	0.1		Within Groups	34.54	79	0.44		
	Hospital	30	3.8	0.61	0.1		Total	40.85	81			
	Total	82	3.6	0.71	0.1							
C2	UDMH	5	4.4	0.82	0.4	C2	Between Groups	5.54	2	2.77	6.8	0.002
	DHN	47	3.5	0.63	0.1		Within Groups	32.39	79	0.41		
	Hospital	30	3.9	0.64	0.1		Total	37.93	81			
	Total	82	3.7	0.68	0.1							
C3	UDMH	5	4.4	0.7	0.3	C3	Between Groups	5.56	2	2.78	6.9	0.002
	DHN	47	3.4	0.62	0.1		Within Groups	31.94	79	0.4		
	Hospital	30	3.8	0.65	0.1			37.5	81			
	Total	82	3.6	0.68	0.1		Total					
C4	UDMH	5	3.9	0.9	0.4	C4	Between Groups	1.14	2	0.57	0.9	0.416
	DHN	47	3.4	0.77	0.1		Within Groups	50.79	79	0.64		
	Hospital	30	3.4	0.83	0.2		Total	51.92	81			
	Total	82	3.4	0.8	0.1							

^*^University Disaster Management Headquarters.

^**^District Health Network.

In contrast, in the other two components, these differences were not significant. Managers with higher education (master’s degree and above) performed better in *Comprehensive risk planning*, and higher education increased their ability to command and lead operational teams (Table 6). Considering that the homogeneity of variances test was significant for the age groups and that these variances showed significant differences across different age groups, the Kruskal-Wallis test was used instead of ANOVA. In this test, it was found that there was a significant difference between the average answers of the managers to the questions in the two components, C2 (*p* = 0.04) and *Support and continuity of service* (C3) (*p* = 0.024).

**Table 6:** Mean distribution and analysis of variance of the difference between the average scores of the questions related to the educational level by four components based on PCA in participants of the incident management system of Zahedan University of Medical Sciences.

Descriptives						ANOVA						
Components	*N*	Mean	Std. deviation	Std. error	Components	Sum of squares	df	Mean square	*F*	Sig.	Components	*N*
C1	Bachelor	46	3.46	0.67	0.1	C1	Between Groups	3.16	1	3.16	6.71	0.011
	MsD and above	36	3.85	0.7	0.12		Within Groups	37.69	80	0.47		
	Total	82	3.63	0.71	0.08		Total	40.85	81			
C2	Bachelor	46	3.57	0.64	0.09	C2	Between Groups	2.49	1	2.49	5.63	0.02
	MsD and above	36	3.92	0.7	0.12		Within Groups	35.44	80	0.44		
	Total	82	3.72	0.68	0.08		Total	37.93	81			
C3	Bachelor	46	3.52	0.6	0.09	C3	Between Groups	1.23	1	1.23	2.70	0.104
	MsD and above	36	3.76	0.76	0.13		Within Groups	36.28	80	0.45		
	Total	82	3.62	0.68	0.08		Total	37.50	81			
C4	Bachelor	46	3.38	0.82	0.12	C4	Between Groups	0.11	1	0.11	0.17	0.686
	MsD and above	36	3.45	0.78	0.13		Within Groups	51.82	80	0.65		
	Total	82	3.41	0.8	0.09		Total	51.92	81			

These findings indicate that managers aged 30–40 years have given higher scores in these two areas than other groups. (Tables 7, 8) Participants with a history of attending the crisis had significantly higher average scores on the questions. In all extracted components, these differences were significant, especially in the first component (C1), which had a significance level of 0.002 (Tables 9, 10).

**Table 7:** The descriptive table of the Kruskal-Wallis test, the difference between the average scores of the questions related to age groups by four components based on PCA in participants of the incident management system of Zahedan University of Medical Sciences.

Components	Age categories	*N*	Mean rank
C1	<=30	8	27.25
	31–40	35	44.66
	> = 41	39	41.59
	Total	82	
C2	<=30	8	21.25
	31–40	35	43.69
	> = 41	39	43.69
	Total	82	
C3	<=30	8	20.31
	31–40	35	45.74
	> = 41	39	42.04
	Total	82	
C4	<=30	8	33.31
	31–40	35	43.09
	> = 41	39	41.76
	Total	82	

**Table 8:** The Kruskal-Wallis test, the difference between the average scores of the questions related to age groups by four components based on PCA in participants of the incident management system of Zahedan University of Medical Sciences.

Components	C1	C2	C3	C4
Kruskal-Wallis H	3.49	6.42	7.48	1.11
df	2	2	2	2
Asymp. Sig.	0.175	0.04	0.024	0.574

**Table 9:** The descriptive table of the Kruskal-Wallis test, the difference between the average scores of the questions related to the history of presence in the crisis according to the four components based on PCA in participants of the incident management system of Zahedan University of Medical Sciences.

Components	History of participating in disaster	*N*	Mean rank
C1	Yes	61	46.3
	No	21	27.57
	Total	82	
C2	Yes	61	46.65
	No	21	26.55
	Total	82	
C3	Yes	61	45.83
	No	21	28.93
	Total	82	
C4	Yes	61	45.11
	No	21	31
	Total	82	

**Table 10:** The Kruskal-Wallis test, the difference between the average scores of the questions related to the history of presence in the crisis according to the four components based on PCA in participants of the incident management system of Zahedan University of Medical Sciences.

Components	C1	C2	C3	C4
Kruskal-Wallis H	9.671	11.139	7.884	5.519
df	1	1	1	1
Asymp. Sig.	0.002	0.001	0.005	0.019

The average answers of the managers to the questions in the four components, according to the different classes of the other investigated variables that were included in the analysis, including the field of study (health, medical, and human sciences), the type of employment (official, contractual and limited-term contract), crisis management training courses, management position (Table 3) and job history (years of working) had no significant difference.

## Discussion

Emergency Operation Centers’ (EOC) primary duties are coordination, communication, allocation and tracking of resources, collection, analysis, and dissemination of information. Also, these centers play an essential role in monitoring the current situation and providing a platform for health professionals to analyze the collected data and perform risk assessments based on it (33). On the other hand, the effective use of EOC and Incident Management System (IMS), especially in countries that face limited resources, has an essential role in political commitment, public participation, accountability, and strategic and operational changes and can be a tool to improve performance and use data for Provide accountability of health workers (16). Empowering EOC members to increase the competencies and self-confidence of the center members has an effective role in the appropriate response to emergencies and improving the efficiency of these centers in response to incidents and disasters (34). The efficiency of these centers leads to a significant difference in the number of injuries and the speed of response to the incident (35).

The present study showed a significant difference between the management level and C1, C2, and C3 scores (Table 5). The significant difference between the scores of EOC managers in university headquarters and EOC managers in health networks and hospitals is related to the scope and complexity of tasks, the amount of coordination required, and the scope of their effects. At the EOC level of the university headquarters, the scope of duties and responsibilities requires more comprehensive planning. Managers in this field should be able to anticipate possible challenges and plan for appropriate responses on a wide scale of society with a strategic approach and accurate knowledge of existing resources and capacities (36). On the other hand, the activities of EOC managers in hospitals and health networks are more focused on specific health crises and risks. Hence, their management framework is more limited and specialized (37).

The continuity of service in the university headquarters’ EOC includes educational, research, and administrative functions, which require more complex management (27). In contrast, the continuity of service in the EOC in hospitals and health networks means providing medical services to patients (38). Bandr Mzahim’s study showed that hospitals focus on solving hospital-specific challenges, including managing mass casualties and epidemics in emergencies and addressing hospital-specific needs, such as patient management, staffing, and medical equipment supply, to ensure the hospital’s delivery of care during a crisis (39). Due to being involved with different groups, including researchers, students, and technical staff, the EOC of the university headquarters needs a higher level of coordination and Command, and to empower these teams, it is required to use different specialties and communicate with related institutions at the national and international level (22). At the same time, the activities of EOC managers in hospitals and health networks are focused on teams providing medical services. The Command aims to facilitate crisis response operations and provide urgent health and medical services (38). It seems that the difference in the average score of the managers working in the EOC of the university headquarters in these three areas compared to the EOC managers of health and treatment networks and hospitals can be explained by the higher complexity and scope of their duties and the need to coordinate with a more significant number of stakeholders.

In components 1 and 2 (C1 and C2), people with higher education (master’s degree and above) scored better. The average score obtained by people with a bachelor’s degree or lower in all four fields was lower than those with a master’s degree or higher (Tables 6, 7). With the increase of specialized knowledge and analytical skills, the ability in *Comprehensive risk planning* and people’s leadership and command skills will increase. These people can better lead operational teams and provide effective crisis management plans. This finding was in line with the results of Xu’s study, which showed that a higher level of education can help improve the ability to plan and make decisions in critical situations (20). David A.L. Coldwell also showed that managers with a high level of education have a better position to predict and prevent crises, analyze complex situations, allocate resources effectively, and use scientific data in their decision-making processes. This educational context leads to the successful management of crises (40). Higher education enhances one’s technical and managerial skills and helps strengthen the skills needed to deal with complex crises.

Based on the study’s findings, people aged 30–40 years presented significantly higher scores in C2 and C3. Also, the average score these people gave in C1 and C4 was higher than other age groups, although these differences were insignificant. In this comparison, the age group below 30 presented lower scores (Tables 7, 8), which can be related to their less experience and less management skills. As individuals progress through chronological stages of development, their cumulative experiential knowledge and cognitive maturation facilitate enhanced decisional efficacy and predictive capabilities in complex, high-stakes environments, potentially optimizing leadership performance and strategic response mechanisms (41).

In their study, Gerry Larsson and Christina Björklund showed that older leaders make more effective decisions using better social skills and experience but still face reduced cognitive abilities in complex situations. Therefore, combining training for young leaders and using the expertise of older leaders can help improve leadership performance in organizations (42).

The present study showed a significant relationship between the experience of past crises and C1, C2, C3, and C4 scores. People who have been present in crises in the past gave higher scores in all the examined areas. This finding was consistent with the results of Shuaib et al.’s study. Previous experience in crisis management can help improve EOC performance in managing health crises and reducing the burden of disease and mortality caused by pandemics (16, 18). On the other hand, it seems that the history of being in previous crises increases the perceived self-efficacy of managers. It is necessary to put the managers in similar crises through training and practice by holding full-scale exercises while familiarizing people with the description of their duties and roles in a crisis, times of crisis, and better preparation in response operations to reduce the effect of company disasters (43, 44).

The systematic collection of information on the current situation, data analysis for risk assessment, and provision of an analytical platform for health professionals expand existing knowledge in crisis management and significantly impact the development and flexibility of health systems (45, 46). Organizational knowledge management, recording and documenting past crises’ experiences, and transferring experiences between different levels by EOCs are essential in promoting managerial knowledge and improving crisis preparedness (46). Encouraging managers to continue their studies at higher levels, holding specialized courses to strengthen analytical and management skills, special training for young managers (under 30 years old), holding full-scale maneuvers to simulate critical situations, using the experiences of experienced managers in training new employees, creating a recording and transferring system of the experiences of past crises, strengthening coordination between different levels, defining detailed job descriptions for each level, appropriate age composition in management teams (using the knowledge of older people and the energy of younger people) and improving decision-making skills in crises can help to improve performance of EOCs (47–50). Training, technology, inter-sectoral cooperation, optimal resource allocation, and the creation of local and national structures are recommended to improve crisis management and increase efficiency in disaster response. Evidence-based training and ongoing exercise programs, such as those conducted by the China Centers for Disease Control (China CDC), can improve staff productivity and coordination in crisis management. The use of digital technologies and the creation of virtual operations centers, which have shown their effectiveness during the COVID-19 pandemic, play an essential role in improving processes and human resource management. Establishing integrated crisis management structures and strengthening cross-sector collaboration, such as Pakistan’s experience with dengue fever, can increase the speed and effectiveness of responses. Improving resource allocation processes and using two-stage systems can reduce response times and improve operational accuracy. Also, establishing emergency operations centers at the local level, similar to the Nigerian experience, would help improve coordination and respond more quickly to health crises (24, 51–54).

## Conclusion

Effective EOCs can reduce pressure on health infrastructure by improving access to resources, reducing response times, and strengthening intersectoral collaboration. As global health threats escalate, this study offers timely insights into optimizing the performance of EOCs to mitigate the impacts of epidemics. By implementing recommendations to improve training, invest in human resources, and intersectoral collaboration, policymakers and health leaders can enhance the effectiveness of EOCs and strengthen their capacity to manage major health crises. Emergency Operations Centers (EOCs) play a crucial role in coordinating and improving the effectiveness of health crisis responses during infectious pandemics. The efficiency of EOCs in their core functions, including resource coordination, strategic communication, data analysis, and follow-up actions, is crucial in improving preparedness and response, leading to increased resilience of health systems and the promotion of public health in emergencies. The results of this study emphasize that investing in the development and empowerment of EOCs, primarily through applying past knowledge and experiences and holding regular exercises, will strengthen organizational responses and, consequently, reduce the harm caused by health crises. It is suggested that future research should examine the long-term impacts of these centers in response to pandemics in greater detail, providing a basis for more sustainable policymaking in managing future crises. The evidence presented in this study should inform global health systems in developing Emergecy Operations Centers (EOCs) for epidemic response. Future research is recommended to investigate the long-term effectiveness of EOCs, informing evidence-based improvements in epidemic preparedness and response strategies.

## Limitations

Sampling was designed as a census, but out of 110 people invited to complete the questionnaire, only 82 (74.5%) responded, which was a limitation of this study. Due to resource limitations, our study was conducted at Zahedan University of Medical Sciences, which may limit the generalizability of the results. Also, due to resource limitations, it was not possible to conduct face-to-face interviews with the samples, and the questionnaires were completed in a self-reported manner. To the extent possible, and with the necessary training over the phone, efforts were made to minimize information bias and selection bias.

## Data Availability

The original contributions presented in the study are included in the article/supplementary material, further inquiries can be directed to the corresponding author.
